# The association between weight-adjusted-waist index and muscle strength in adults: a population-based study

**DOI:** 10.3389/fpubh.2024.1429244

**Published:** 2024-07-26

**Authors:** Lihan Xu, Haojing Zhou

**Affiliations:** ^1^School of Stomatology, Hangzhou Normal University, Hangzhou, Zhejiang, China; ^2^The First School of Clinical Medicine, Zhejiang Chinese Medical University, Hangzhou, Zhejiang, China

**Keywords:** cross-sectional studies, muscle strength, NHANES, obesity, sarcopenia, weight-adjusted-waist index

## Abstract

**Background:**

The relationship between the weight-adjusted-waist index (WWI) and grip strength, a crucial marker in assessing sarcopenia, lacks clarity. We aimed to explore the relationship between WWI and muscle strength across genders.

**Methods:**

The cross-sectional study involved adults with complete data on WWI and grip strength from the 2011–2014 National Health and Nutrition Examination Survey. WWI was derived by dividing waist circumference by the square root of weight. Weighted multivariable logistic regression and smooth curve fitting techniques were used to examine the independent association and potential non-linear relationship between WWI and grip strength. A two-piecewise linear regression model was utilized to determine the threshold effect. Additionally, subgroup analyses and interaction tests were conducted.

**Results:**

The study encompassed 9,365 participants, including 4,661 males and 4,704 females. Multivariate logistic regression analysis revealed a negative correlation between WWI and grip strength among males (*β* = −11.49, 95% CI: −12.38, −10.60, *p* < 0.001) as well as females (*β* = −2.53, 95% CI: −2.98, −2.08, *p* < 0.001). Subgroup analysis showed that the negative correlation of WWI with grip strength remained consistent across various age groups and levels of obesity for both males and females.

**Conclusion:**

An increase in WWI correlates with reduced muscle strength in both males and females. WWI was negatively associated not only with muscle mass but also with muscle strength. WWI may serve as an assessment tool for sarcopenia, but further large-scale studies are needed to clarify causality.

## 1 Background

Sarcopenia is delineated as the gradual and progressive reduction in skeletal muscle mass and strength, often accompanied by a decline in physical function (1, 2). This condition stands as a significant aging-related syndrome, independently foretelling multiple clinically consequential adverse outcomes, encompassing an elevated susceptibility to fractures, reduced quality of life, impaired mobility, and heightened mortality rates (3–5). In Asian countries, the prevalence of sarcopenia varies between 5.5 and 25.7%, with a higher occurrence among males (5.1–21.0% in male compared to 4.1–16.3% in female), leading to significant socioeconomic implications (6, 7). Throughout the revision of sarcopenia guidelines, there is an increased emphasis on muscle strength. This shift stems from the acknowledgment that, in anticipating negative outcomes, muscle strength surpasses muscle mass in predictive accuracy (1, 8). Grip strength, acknowledged as a dependable proxy for overall muscle strength, has garnered considerable attention in numerous guidelines as a pivotal marker for assessing and diagnosing sarcopenia (1, 7, 9).

The weight-adjusted-waist index (WWI) is a recently introduced anthropometric measure derived by standardizing waist circumference (WC) to body weight, computed as the WC in centimeters divided by the square root of weight in kilograms (10). Similar to body mass index (BMI), a higher WWI score signifies increased levels of obesity. Previous studies demonstrated an independent association between WWI and sarcopenic obesity in specific populations like type 2 diabetes mellitus patients and males undergoing maintenance hemodialysis (11, 12). Notably, WWI exhibits a stronger correlation with sarcopenic obesity in older men compared to other anthropometric indices such as waist-to-height ratio, BMI, and WC (13).

Previous studies have demonstrated an inverse correlation between WWI and both appendicular lean mass and abdominal muscle mass among middle-aged and older adult populations (14–16). The association between WWI and sarcopenia has been initially explored. However, the association between WWI and grip strength, a crucial component in assessing sarcopenia, lacks clarity.

The objectives of this study are as follows: Firstly, it aims to investigate the relationship between WWI and grip strength in the adult population. Secondly, the study aims to explore the potential of WWI as a predictive indicator for sarcopenia. It is assumed that there exists a negative correlation between WWI and grip strength.

## 2 Methods

### 2.1 Data source and study population

Data were sourced from National Health and Nutrition Examination Survey (NHANES), a nationally conducted cross-sectional survey aimed at gathering information on potential health risk factors and the nutritional status of non-institutionalized civilians in the United States, conducted by the National Center for Health Statistics. A complex, stratified, multistage probability cluster sampling design was employed to obtain a representative sample of the entire the United States population (17). The NHANES study protocols were sanctioned by the Research Ethics Review Board of the NCHS. Written informed consent was acquired from all survey participants or from a parent/legal guardian for those under 16 years of age. Comprehensive details regarding the NHANES study design and data can be accessed publicly at https://www.cdc.gov/nchs/nhanes/.

**Figure 1:**
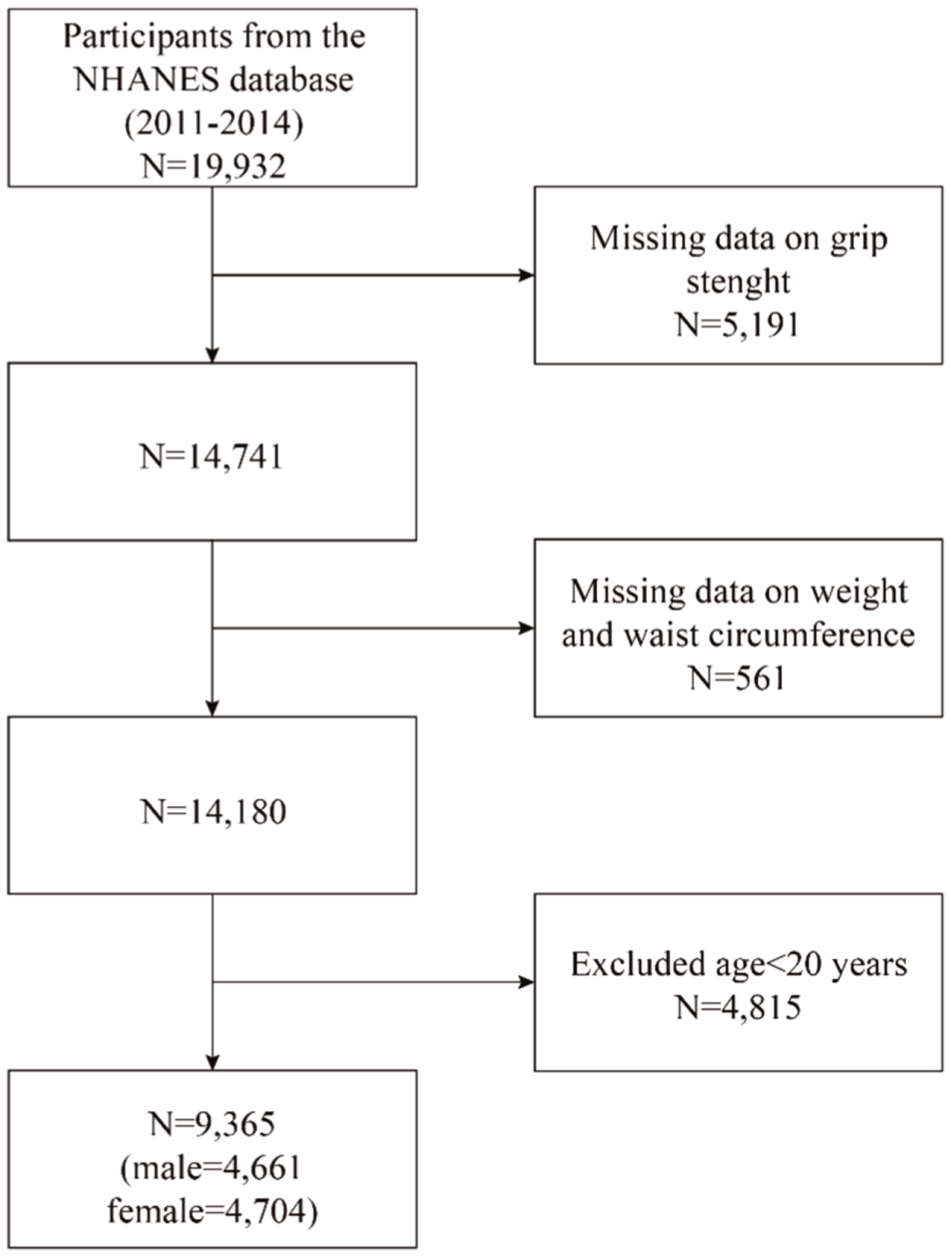
Flowchart of population included in our final analysis.

Our study utilized data from the NHANES survey cycles spanning 2011 to 2014, as only these cycles encompassed information on grip strength and WWI. Initially, 19,932 participants were enrolled; however, following the exclusion of those lacking grip strength data (*n* = 5,191), missing WWI data (*n* = 561), and those under 20 years of age (*n* = 4,815), our final analysis comprised 9,365 participants. The cohort consisted of 4,661 males and 4,704 females (Figure 1).

### 2.2 Assessment of WWI

The WWI, an anthropometric index combining WC and weight, serves as an estimation tool for obesity. A higher WWI score indicates a higher level of obesity. Body measurement data pertaining to WC and weight were gathered within the mobile examination center by proficient health technicians. The WWI for each participant was computed as the WC in centimeters divided by the square root of weight in kilograms, rounded to two decimal places. In our analysis, we regarded WWI as a continuous variable, subsequently grouping participants according to WWI quartiles for further investigation. WWI was utilized as an exposure variable in our study.

### 2.3 Assessment of grip strength

Grip strength measurements were conducted during the NHANES 2011–2014 survey, following the protocol specified for that period. The assessment of grip strength adhered to the guidelines outlined in the Muscle Function Procedures Manual and utilized the Takei Digital Grip Strength Dynamometer, Model T.K.K.5401 (Takei Scientific Instruments Co., Niigata, Japan). Beforehand, the dynamometer was adjusted to fit each participant’s hand size while they stood with their arm straight down and wrist in a neutral position. Participants were instructed to exert maximum force while squeezing the dynamometer using one hand, repeating the test three times on alternate hands with a 60-s interval between measurements of the same hand. The NHANES recorded the combined handgrip strength by summing the highest readings from each hand, expressed in kilograms.

### 2.4 Covariates

Our study incorporated covariates that might influence the relationship between WWI and grip strength. These included gender (male/female), age (year), race (non-Hispanic White/non-Hispanic Black/Mexican American/other races), education level (less than high school/high school/more than high school), height (cm), weight (kg), BMI (kg/m^2^), WC (cm), intake of energy (kcal/day), intake of protein (gm/day), smoking status (yes/no), alcohol status (yes/no), hypertension (yes/no), diabetes (yes/no) and hypercholesterolemia (yes/no). Energy and protein intake were calculated by averaging the intake across day 1 and day 2. Smoking status was determined based on whether one had smoked at least 100 cigarettes in life. Participants who had at least 12 alcohol drinks per year were considered drinkers. Hypertension, diabetes, and hypercholesterolemia were identified based on self-reported diagnoses of these conditions. Subgroup analysis categorized BMI as <25, 25–29.9, and ≥30 kg/m^2^, representing normal weight, overweight, and obese categories, respectively, for the participants. Further details were accessible at www.cdc.gov/nchs/nhanes.

### 2.5 Statistical analysis

The statistical analyses adhered to Centers for Disease Control and Prevention guidelines, and an adjusted NHANES sampling weight was utilized, considering the intricate multistage cluster survey design during analysis. Significant disparities in grip strength were observed between male and female (18). Consequently, this study was segregated into male and female groups to investigate the correlation between WWI and grip strength. Continuous variables were expressed as mean ± standard deviation, while categorical variables were displayed as percentages. Group differences based on WWI quartiles were assessed using either a weighted Student’s *t*-test (for continuous variables) or a weighted chi-square test (for categorical variables). Three models of multivariable logistic regression were employed to assess the association between WWI and grip strength. In model 1, no covariates were adjusted. In model 2, age, race, and education level were adjusted. Model 3 was adjusted for age, race, education level, BMI, intake of energy, intake of protein, smoking status, alcohol status, hypertension, diabetes, and hypercholesterolemia. Subgroup analyses examining the associations between WWI and grip strength were performed, stratified by gender (male/female), age groups (20–39/40–59/≥60 years), and BMI categories (normal weight/overweight/obesity). These stratified factors were considered as predetermined potential effect modifiers. An interaction term was introduced to assess the diversity of associations among the subgroups. Additionally, smooth curve fittings were employed to identify potential non-linear relationships between WWI and grip strength, and a two-piecewise linear regression model was employed to further explore their threshold effects. Missing values for continuous variables were imputed using the mean, and for categorical variables, the mode was used, limited to existing cases of those variables. All analyses were performed using R version 3.4.3 (http://www.R-project.org, The R Foundation) and Empower software (www.empowerstats.com; X&Y solutions, Inc., Boston, MA). Statistical significance was set at a two-sided *p*-value <0.05.

## 3 Results

### 3.1 Baseline characteristics of participants

The demographic characteristics of the study cohort categorized by gender-specific quartiles of WWI were presented in Tables 1, 2. Among males, the mean WWI was 10.82 ± 0.81, with quartile ranges of 8.37–10.25, 10.25–10.82, 10.82–11.39, and 11.39–14.79 for Quartiles 1 through 4, respectively. Grip strength exhibited a mean of 87.23 ± 18.92 kg, with values of 95.79 ± 17.45, 90.86 ± 16.98, 85.30 ± 17.62, and 76.98 ± 18.29 kg for Quartiles 1, 2, 3, and 4, respectively. Among females, the mean WWI was 11.22 ± 0.83, with quartile ranges of 8.38–10.63, 10.63–11.20, 11.20–11.79, and 11.79–14.20 for Quartiles 1 through 4, respectively. Grip strength exhibited a mean of 55.63 ± 11.92 kg, with values of 59.52 ± 10.64, 57.33 ± 11.39, 54.85 ± 11.55, and 50.84 ± 12.27 kg for Quartiles 1, 2, 3, and 4, respectively.

**Table 1:** Basic characteristics of participants by WWI quartile in male.

Characteristics	Overall	Weight-adjusted-waist index quartile (cm/√kg)				*P*-value
	*N* = 4,661	Q1 (8.37–10.25) *N* = 1,165	Q2 (10.25–10.82) *N* = 1,165	Q3 (10.82–11.39) *N* = 1,165	Q4 (11.39–14.79) *N* = 1,166	
Age (years)	47.93 ± 17.53	34.78 ± 13.08	44.44 ± 14.71	52.32 ± 15.75	60.18 ± 15.47	<0.001
Race/ethnicity, *N* (%)						<0.001
Non-Hispanic White	1898(40.72)	409 (35.11)	444 (38.11)	457 (39.23)	588 (50.43)	
Non-Hispanic Black	1,086(23.30)	416 (35.71)	251 (21.55)	238 (20.43)	181 (15.52)	
Mexican American	543(11.65)	58 (4.98)	154 (13.22)	161 (13.82)	170 (14.58)	
Other race	1,134(24.33)	282 (24.21)	316 (27.12)	309 (26.52)	227 (19.47)	
Education level, *N* (%)						<0.001
Less than high school	1,044(22.41)	182 (15.62)	219 (18.80)	309 (26.55)	334 (28.67)	
High school	1,073(23.03)	254 (21.80)	267 (22.92)	281 (24.14)	271 (23.26)	
More than high school	2,542(54.56)	729 (62.58)	679 (58.28)	574 (49.31)	560 (48.07)	
Height (cm)	174.38 ± 7.71	177.41 ± 7.32	175.24 ± 7.40	173.43 ± 7.45	171.45 ± 7.41	<0.001
Weight (kg)	86.49 ± 20.60	76.83 ± 14.60	84.50 ± 17.72	88.54 ± 19.46	96.06 ± 24.46	<0.001
Body Mass Index (kg/m^2^)	28.34 ± 6.02	24.31 ± 3.77	27.37 ± 4.56	29.26 ± 5.18	32.43 ± 6.96	<0.001
Waist Circumference (cm)	100.23 ± 15.97	85.34 ± 9.14	96.53 ± 10.12	103.86 ± 11.32	115.19 ± 15.27	<0.001
Intake of Energy (kcal/day)	2371.50 ± 904.05	2614.61 ± 1018.46	2412.32 ± 859.36	2297.28 ± 868.31	2161.95 ± 794.64	<0.001
Intake of Protein (gm/day)	94.85 ± 39.99	104.28 ± 47.56	96.98 ± 37.76	91.57 ± 36.65	86.56 ± 34.57	<0.001
Smoking status, *N* (%)						<0.001
YES	2,428(52.13)	521 (44.72)	591 (50.77)	625 (53.74)	691 (59.26)	
NO	2,230(47.87)	644 (55.28)	573 (49.23)	538 (46.26)	475 (40.74)	
Alcohol status, *N* (%)						0.129
YES	3,718(84.34)	906 (83.81)	952 (86.47)	919 (82.94)	941 (84.17)	
NO	690(15.65)	175 (16.19)	149 (13.53)	189 (17.06)	177 (15.83)	
Hypertension, *N* (%)						<0.001
YES	1,604(34.41)	178 (15.31)	317 (27.23)	488 (41.89)	621 (53.35)	
NO	3,052(65.48)	985 (84.69)	847 (72.77)	677 (58.11)	543 (46.65)	
Diabetes, *N* (%)						<0.001
YES	685(14.70)	38 (3.26)	103 (8.85)	192 (16.48)	352 (30.19)	
NO	3,975(85.30)	1,127 (96.74)	1,061 (91.15)	973 (83.52)	814 (69.81)	
Hypercholesterolemia, *N* (%)						<0.001
YES	1,627(35.13)	180 (15.49)	355 (30.71)	500 (43.18)	592 (51.26)	
NO	3,004(64.87)	982 (84.51)	801 (69.29)	658 (56.82)	563 (48.74)	
WWI (cm/√kg)	10.82 ± 0.81	9.77 ± 0.37	10.55 ± 0.16	11.10 ± 0.17	11.84 ± 0.38	<0.001
Grip strength (kg)	87.23 ± 18.92	95.79 ± 17.45	90.86 ± 16.98	85.30 ± 17.62	76.98 ± 18.29	<0.001

Continuous variables were presented as mean ± SD, and the *P*-value was derived using a weighted linear regression model, Categorical variables were presented as percentages, and the *P*-value was derived through a weighted chi-square test. WWI, weight-adjusted-waist index.

**Table 2:** Basic characteristics of participants by WWI quartile in female.

Characteristics	Overall	Weight-adjusted-waist index quartile (cm/√kg)				*P*-value
	*N* = 4,704	Q1 (8.38–10.63) *N* = 1,176	Q2 (10.63–11.20) *N* = 1,176	Q3 (11.20–11.79) *N* = 1,176	Q4 (11.79–14.20) *N* = 1,176	
Age (years)	48.32 ± 17.26	38.47 ± 14.63	46.20 ± 15.94	51.81 ± 16.00	56.81 ± 16.80	<0.001
Race/ethnicity, *N* (%)						<0.001
Non-Hispanic White	1925(40.92)	499 (42.43)	479 (40.73)	432 (36.73)	515 (43.79)	
Non-Hispanic Black	1,098(23.34)	301 (25.60)	281 (23.89)	296 (25.17)	220 (18.71)	
Mexican American	521(11.08)	75 (6.38)	102 (8.67)	168 (14.29)	176 (14.97)	
Other race	1,160(24.66)	301 (25.60)	314 (26.70)	280 (23.81)	265 (22.53)	
Education level, *N* (%)						<0.001
Less than high school	926(19.69)	116 (9.86)	188 (16.00)	270 (22.96)	352 (29.93)	
High school	957(20.35)	181 (15.39)	213 (18.13)	286 (24.32)	277 (23.55)	
More than high school	2,820(59.96)	879 (74.74)	774 (65.87)	620 (52.72)	547 (46.51)	
Height (cm)	160.79 ± 7.19	164.21 ± 6.71	161.40 ± 6.54	159.73 ± 6.67	157.80 ± 7.26	<0.001
Weight (kg)	76.17 ± 20.90	67.17 ± 15.89	73.72 ± 18.67	79.02 ± 21.21	84.78 ± 22.97	<0.001
Body Mass Index (kg/m^2^)	29.40 ± 7.55	24.84 ± 5.35	28.16 ± 6.35	30.80 ± 7.27	33.81 ± 7.91	<0.001
Waist Circumference (cm)	97.35 ± 16.80	82.77 ± 10.34	93.15 ± 11.68	101.35 ± 13.29	112.13 ± 15.62	<0.001
Intake of Energy (kcal/day)	1783.42 ± 662.25	1878.92 ± 735.12	1786.23 ± 612.91	1764.28 ± 649.81	1704.28 ± 633.49	<0.001
Intake of Protein (gm/day)	69.36 ± 27.73	72.56 ± 29.51	69.84 ± 26.78	68.70 ± 27.76	66.36 ± 26.45	<0.001
Smoking status, *N* (%)						<0.001
YES	1,625(34.56)	341 (29.05)	398 (33.84)	416 (35.37)	470 (39.97)	
NO	3,077(65.44)	833 (70.95)	778 (66.16)	760 (64.63)	706 (60.03)	
Alcohol status, *N* (%)						<0.001
YES	2,763(63.11)	785 (72.55)	698 (64.33)	677 (61.60)	603 (54.23)	
NO	1,615(36.89)	297 (27.45)	387 (35.67)	422 (38.40)	509 (45.77)	
Hypertension, *N* (%)						<0.001
YES	1,695(36.06)	194 (16.51)	353 (30.04)	505 (42.98)	643 (54.72)	
NO	3,005(63.94)	981 (83.49)	822 (69.96)	670 (57.02)	532 (45.28)	
Diabetes, *N* (%)						<0.001
YES	670(14.26)	42 (3.57)	97 (8.26)	202 (17.18)	329 (28.02)	
NO	4,029(85.74)	1,133 (96.43)	1,077 (91.74)	974 (82.82)	845 (71.98)	
Hypercholesterolemia, *N* (%)						<0.001
YES	1,577(33.71)	199 (17.02)	340 (29.01)	477 (40.63)	561 (48.24)	
NO	3,101(66.29)	970 (82.98)	832 (70.99)	697 (59.37)	602 (51.76)	
WWI (cm/√kg)	11.22 ± 0.83	10.16 ± 0.36	10.93 ± 0.16	11.49 ± 0.17	12.28 ± 0.40	<0.001
Grip strength (kg)	55.63 ± 11.92	59.52 ± 10.64	57.33 ± 11.39	54.85 ± 11.55	50.84 ± 12.27	<0.001

Continuous variables were presented as mean ± SD, and the *P*-value was derived using a weighted linear regression model, Categorical variables were presented as percentages, and the *P*-value was derived through a weighted chi-square test. WWI, weight-adjusted-waist index.

### 3.2 The association between WWI and grip strength

Table 3 illustrated the relationship between WWI and grip strength in males and females using three weighted generalized linear regression models. In the fully adjusted model (Model III), a negative correlation between WWI and grip strength was evident in males (*β* = −11.49, 95% CI: −12.38, −10.60, *p* < 0.001). Similarly, females displayed a negative correlation between WWI and grip strength (*β* = −2.53, 95% CI: −2.98, −2.08, *p* < 0.001). Regardless of gender, compared to the lowest quartile of WWI, the second, third, and fourth quartiles of WWI exhibited a statistically significant association with grip strength (Male: Q2: *β* = −4.24, 95% CI: −5.59, −2.89, *p* < 0.001; Q3: *β* = −9.35, 95% CI: −10.91, −7.80, *p* < 0.001; Q4: *β* = −19.84, 95% CI: −21.72, −17.96, *p* < 0.001; Female: Q2: *β* = −1.25, 95% CI: −2.06, −0.45, *p* = 0.002; Q3: *β* = −2.33, 95% CI: −3.22, −1.45, *p* < 0.001; Q4: *β* = −5.18, 95% CI: −6.18, −4.18, *p* < 0.001). Sensitivity analysis confirmed this trend (*p* for trend <0.001).

**Table 3:** The associations between WWI and grip strength.

	Model 1 β (95% CI) *p*-value	Model 2 β (95% CI) *p*-value	Model 3 β (95% CI) *p*-value
Male			
WWI (continuous)	−8.29 (−8.90, −7.67) <0.001	−4.04 (−4.77, −3.32) <0.001	−11.49 (−12.38, −10.60) <0.001
WWI (quartile)			
Quartile 1	Reference	Reference	Reference
Quartile 2	−3.52 (−4.87, −2.17) <0.001	0.66 (−0.68, 1.99) 0.336	−4.24 (−5.59, −2.89) <0.001
Quartile 3	−8.53 (−9.90, −7.15) <0.001	−1.40 (−2.85, 0.04) 0.057	−9.35 (−10.91, −7.80) <0.001
Quartile 4	−16.87 (−18.27, −15.47) <0.001	−7.35 (−8.93, −5.77) <0.001	−19.84 (−21.72, −17.96) <0.001
P for trend	<0.001	<0.001	<0.001
Female			
WWI (continuous)	−3.58 (−3.96, −3.19) <0.001	−0.81 (−1.19, −0.43) <0.001	−2.53 (−2.98, −2.08) <0.001
WWI (quartile)			
Quartile 1	Reference	Reference	Reference
Quartile 2	−2.42 (−3.28, −1.56) <0.001	0.11 (−0.67, 0.89) 0.783	−1.25 (−2.06, −0.45) 0.002
Quartile 3	−4.26 (−5.14, −3.37) <0.001	−0.03 (−0.86, 0.80) 0.942	−2.33 (−3.22, −1.45) <0.001
Quartile 4	−7.77 (−8.68, −6.87) <0.001	−1.84 (−2.72, −0.97) <0.001	−5.18 (−6.18, −4.18) <0.001
*P* for trend	<0.001	<0.001	<0.001

Model 1: no covariates were adjusted. Model 2: age, race, and education level were adjusted. Model 3: age, race, education level, body mass index, intake of energy, intake of protein, smoking status, alcohol status, hypertension, diabetes, and hypercholesterolemia were adjusted. WWI, weight-adjusted-waist index.

### 3.3 Subgroup analysis

To investigate the relationship between WWI and grip strength in the general population, subgroup analyses and interaction tests were conducted based on age and BMI for both males and females (Table 4). In males, the negative correlation between WWI and grip strength endured across all age groups. When stratified by BMI, the negative relationship between WWI and grip strength remained consistent across the normal weight, overweight, and obese populations. Similarly, in females, the negative correlation between WWI and grip strength was stable across different age groups and levels of obesity. Interaction analyses indicated that in females, the association between WWI and grip strength was influenced by BMI rather than age, while in males, neither age nor BMI appeared to affect this association.

**Table 4:** Subgroup analysis of the association between WWI and grip strength.

Subgroup	*β* (95% CI)	*P*-value	*P* for interaction
Male			
Age			0.506
20–39 years	−12.13 (−13.49, −10.76)	<0.001	
40–59 years	−11.33 (−12.71, −9.96)	<0.001	
60–80 years	−12.50 (−14.04, −10.96)	<0.001	
BMI			0.819
Normal weight	−9.66 (−11.31, −8.01)	<0.001	
Overweight	−10.15 (−11.51, −8.80)	<0.001	
Obese	−10.32 (−11.64, −9.01)	<0.001	
Female			
Age			0.702
20–39 years	−2.72 (−3.46, −1.98)	<0.001	
40–59 years	−2.98 (−3.73, −2.24)	<0.001	
60–80 years	−3.17 (−3.95, −2.39)	<0.001	
BMI			0.029
Normal weight	−2.81 (−3.62, −2.00)	<0.001	
Overweight	−3.06 (−3.90, −2.22)	<0.001	
Obese	−1.78 (−2.43, −1.13)	<0.001	

Age, race, education level, body mass index, intake of energy, intake of protein, smoking status, alcohol status, hypertension, diabetes, and hypercholesterolemia were adjusted. BMI, Body Mass Index.

### 3.4 Non-linear relationship between WWI and grip strength

**Figure 2:**
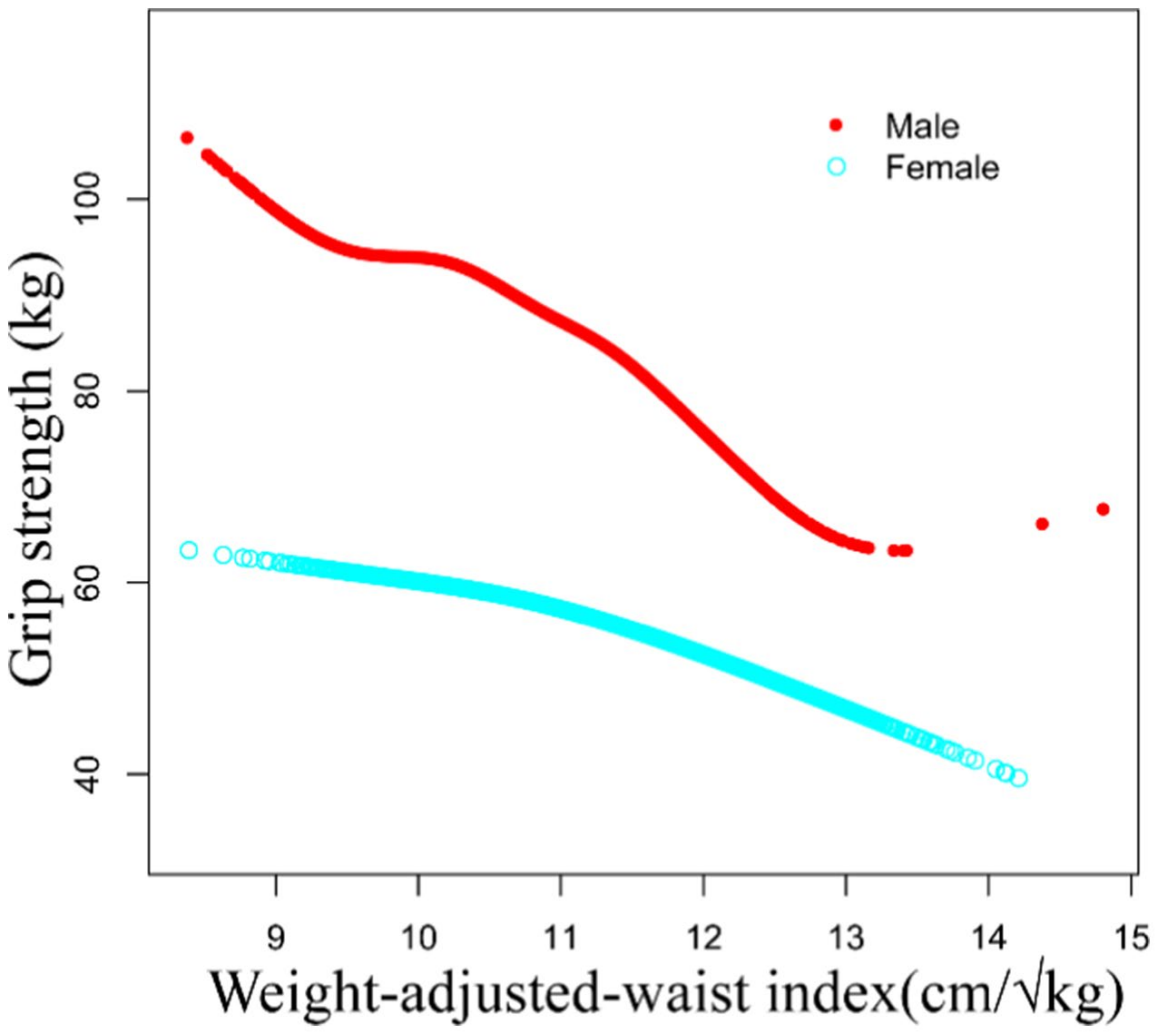
The non-linear associations between WWI and grip strength.

As depicted in Figure 2, a non-linear inverse relationship between WWI and grip strength was observed in both males and females. Table 5 illustrated that the inflection points (K), determined through the two-piecewise linear regression model, were calculated at 10.83 for males and 11.43 for females. Both genders exhibited a significant negative correlation between WWI and grip strength on either side of the inflection point. Among males, on the left side of the inflection point, a unit increased in WWI corresponded to a reduction of grip strength by 7.59 kg, while on the right side, each unit rose in WWI resulted in a decrease of grip strength by 15.16 kg. Similarly, among females, on the left side of the inflection point, a unit increased in WWI corresponded to a reduction of grip strength by 1.45 kg, and on the right side, each unit rose in WWI led to a decrease of grip strength by 4.18 kg. Both groups exhibited logarithmic likelihood ratio test *p*-values <0.001.

**Table 5:** Threshold effect analysis of WWI on grip strength using a two-piecewise linear regression model.

	Fitting by the standard linear model	Fitting by the two-piecewise linear model			
		Inflection point (K)	<K-segment effect	>K-segment effect	Log likelihood ratio
Male					
WWI	−11.49 (−12.38, −10.60) <0.001	10.83	−7.59 (−8.90, −6.27) <0.001	−15.16 (−16.43, −13.88) <0.001	<0.001
Female					
WWI	−2.53 (−2.98, −2.08) <0.001	11.43	−1.45 (−2.10, −0.79) <0.001	−4.18 (−5.03, −3.32) <0.001	<0.001

Age, race, education level, body mass index, intake of energy, intake of protein, smoking status, alcohol status, hypertension, diabetes, and hypercholesterolemia were adjusted. WWI, weight-adjusted-waist index.

## 4 Discussion

The objective of this study was to assess the correlation between WWI and grip strength. In our cross-sectional analysis encompassing 4,661 male and 4,704 female, a notable adverse correlation between WWI and grip strength was observed. This correlation persisted even subsequent to adjustments for all potential confounding variables. Subgroup analysis showed that the negative correlation of WWI with grip strength remained consistent across various age groups and levels of obesity for both males and females. Our findings indicated a negative association between WWI and grip strength in the adult population of the United States, which held true across various age groups and levels of obesity.

To our knowledge, this study was the inaugural exploration into the association between WWI and muscle strength. The diagnostic criteria for sarcopenia, as proposed by the European Working Group on Sarcopenia in Older People, comprise three elements: low muscle strength, low physical performance and low muscle mass (1). Prior investigations have shown an inverse relationship between WWI and both abdominal muscle mass and appendicular lean mass in middle-aged and older adult cohorts (14–16). However, no research has explicitly elucidated the correlation between WWI and muscle strength which is more associated with poor outcomes. This study filled this gap. Similar to muscle mass, our findings revealed a negative correlation between WWI and muscle strength.

The association between WWI and bone and muscle mass has been partially substantiated. Studies indicated a negative correlation between WWI and bone mineral density in the lumbar spine, pelvis, femoral neck, and total (19). And, previous research on WWI and muscle mass primarily concentrated on middle-aged and older adult individuals (14–16) and even people with characteristic diseases (11, 12), and noted gender disparities. However, through subgroup analysis, the correlation between WWI and muscle strength remained consistent across young, middle-aged, and older adults, encompassing both males and females. BMI stratification indicated that the negative correlation between WWI and grip strength applies consistently across different levels of obesity. The prevalence of obesity is rising worldwide (20). Data sourced from the World Health Organization indicates that 39% of the global adult population are overweight, of which 13% fall under the category of obesity, with a persistent increase in the count of obese individuals (21, 22). The relationship between WWI and grip strength also existed in the obese population. Given this context, WWI shows a growing potential to predict muscle strength.

The association between other obesity indices (BMI, body fat rate, waist-to-hip ratio) and muscle strength remained contentious. In several studies, conflicting results have emerged. Agtuahene et al. (23) showed a positive link between BMI and grip strength in youth, while Alahmari et al. (24) suggested no correlation between BMI and grip strength in grown men. Moreover, Siqueira revealed no substantial relationship between BMI and leg strength in active older adult individuals, except concerning grip strength (25). The sample sizes in these studies were relatively small, ranging from 64 to 304 participants. Nevertheless, a substantial cross-sectional study with a sample size exceeding 7,000 middle-aged individuals uncovered a positive association between BMI and grip strength (26). Variations in sample sizes and age group differences among the studies might explain these inconsistencies in research findings. In the context of body fat percentage, a comprehensive study revealed a positive correlation between body fat percentage and grip strength among middle-aged participants (26). Conversely, a separate study demonstrated a negative correlation between body fat percentage and abdominal strength. Notably, there was no observed correlation between waist-to-hip ratio and either grip strength or abdominal strength (26, 27).

However, as an index indicating increased obesity with higher values, BMI either lacks correlation or shows a positive one with muscle strength. Conversely, this study illustrated that WWI, also employed as an obesity indicator, displayed a negative correlation with muscle strength. The relationship between obesity and muscle is intricate. In the progression of obesity and muscle mass decline, a phenomenon of fat redistribution occurs. This presents as the transfer of fat from subcutaneous regions to the abdominal cavity (visceral fat) and its infiltration into muscles (28–30). This results in diminished muscle strength and functionality. Fat infiltration into muscles heightens the risk of advancing toward obesity, while obesity hampers muscle regeneration, initiating pre-sarcopenia. This synergistic interaction between muscle loss and fat infiltration could initiate and worsen sarcopenic obesity. While valuable for illustrating trends in obesity prevalence at a population level, BMI offers a rudimentary assessment of overall adiposity. Because BMI cannot differentiate between fat and muscle mass, the ratio of lean mass to fat mass may differ despite similar BMI values (31, 32). The reduction in muscle mass leading to weight loss is offset by an augmentation in visceral fat, leading to a circumstance wherein, despite diminished muscle strength, the BMI shows no alteration. This observation was also employed to elucidate the phenomenon of the obesity paradox (28, 31, 33). Additionally, the relocation of fat from subcutaneous to visceral areas does not necessarily imply weight gain, but it may enlarge WC (34), thus increasing WWI while BMI remains unchanged. This might be the reason behind the inconsistency in the correlation between WWI and BMI, two obesity indicators, with muscle strength.

The SARC-F scale is utilized for the identification of individuals potentially affected by sarcopenia. However, its sensitivity is limited, frequently resulting in the omission of certain patients (35). And the suggested techniques for assessing muscle strength and mass necessitate specialized tools such as computed tomography, magnetic resonance imaging, and ultrasound (1). Nevertheless, these methods entail substantial expenses and time commitments. The estimation of muscle strength and muscle mass without such specialized tools poses a challenge, and presently, no anthropometric index adequately reflects both these parameters. Previous studies and this study have demonstrated that WWI is correlated with both muscle strength and muscle mass. As a result, WWI holds promise as a simple screening tool for identifying sarcopenia or to complement the SARC-F scale for better detection of sarcopenia cases.

The study possesses several strengths. Primarily, it relied on NHANES data and conducted analyses while accounting for the appropriate NHANES sample weights. Secondly, we meticulously adjusted confounding covariates, enhancing the reliability of our results and their applicability to a broader spectrum of individuals. Nevertheless, the study also presents certain limitations. Initially, owing to the cross-sectional design, establishing a causal relationship was unattainable. Therefore, further prospective studies involving larger sample sizes remain imperative to elucidate causality. Furthermore, despite our adjustment for several potential covariates, the influence of other conceivable confounding factors could not be entirely ruled out.

## 5 Conclusion

Increased WWI correlated with reduced muscle strength in both male and female. As the WWI index rises, the strength of this negative correlation intensifies. The negative correlation of WWI with grip strength remained consistent across various age groups and levels of obesity. WWI may serve as an assessment tool for sarcopenia. Nonetheless, additional large-scale prospective studies are required to elucidate the precise causal link in this association.

## Data availability statement

The original contributions presented in the study are included in the article/supplementary material, further inquiries can be directed to the corresponding author.

## Ethics statement

The studies involving humans were approved by Research Ethics Review Board of the National Center for Health Statistics. The studies were conducted in accordance with the local legislation and institutional requirements. The participants provided their written informed consent to participate in this study.

## Author contributions

LX: Conceptualization, Data curation, Investigation, Methodology, Software, Supervision, Writing – original draft, Writing – review & editing. HZ: Formal analysis, Funding acquisition, Project administration, Resources, Validation, Visualization, Writing – original draft, Writing – review & editing.
